# TOZAL Study: An open case control study of an oral antioxidant and omega-3 supplement for dry AMD

**DOI:** 10.1186/1471-2415-7-3

**Published:** 2007-02-26

**Authors:** Francis E Cangemi

**Affiliations:** 1 Vitreo-Retinal Associates of New Jersey, 119 Prospect Street, Ridgewood, New Jersey 07450 USA

## Abstract

**Background:**

The primary objective of this prospective study was to measure the change from baseline in visual function – Best-Corrected Visual Acuity (BCVA) via the Early Treatment Diabetic Retinopathy Study (ETDRS) chart, contrast sensitivity, central 10 degree visual fields and retinal imaging (angiograms and photographs) at 6 months in subjects with atrophic (dry) age-related macular degeneration treated with a targeted nutritional supplement.

**Methods:**

37 mixed gender patients with a mean age of 76.3 +/- 7.8 years were enrolled at 5 independent study sites and received standard of care with a novel formulation of a nutritional supplement. Results were compared to a placebo cohort constructed from the literature that was matched for inclusion and exclusion criteria. A paired t-test was used to test a null hypothesis and a two-sided alpha level of 0.05 was used to determine statistical significance.

**Results:**

76.7% of subjects receiving the nutritional supplement demonstrated stabilization or improvement of BCVA at 6 months. Subjects gained an average of 0.0541 logMAR or one-half of a line of visual acuity (VA) over the 6-month period. There was a statistically significant improvement in VA from baseline with *P *= .045. The results provide strong evidence that the treatment being studied produces an improvement in VA.

**Conclusion:**

Treatment with this unique nutritional supplement increased VA above the expected baseline decrease in the majority of patients in this population with dry macular degeneration. The results of the TOZAL study agree with the LAST and CARMIS studies and are predictive for positive visual acuity outcomes in the AREDS II trial. However, patients will likely require supplementation for longer than 6 months to effect changes in additional visual parameters.

## Background

Age-related Macular Degeneration (AMD) is a progressive disorder associated with central vision loss and is the leading cause of visual impairment and blindness in people over the age of 60. More than 15 million Americans over the age of 60 have AMD with an additional 50 million Americans at risk for developing the disorder [1]. Dry, atrophic, or non-exudative, AMD is the most common form and is characterized by progressive devitalization of retinal pigment epithelium (RPE) and the formation of fatty deposits under the RPE known as soft drusen [1]. Although the underlying cause of AMD is unknown, risk factors have been defined and include age greater than 50, Caucasian race, nutrition, smoking, atherosclerotic vascular disease, genetics, and sunlight exposure [1-4]. At this time, there is no known cure for AMD. Patients not receiving treatment have demonstrated a loss of VA at 6 months of at least 0.8 lines and up to 1.5 lines [5-7].

Multiple studies have suggested that manipulation of nutritional factors can play a significant role in slowing the onset or limiting the effects of AMD [8-13]. In 1996, Richer et al found that a broad-spectrum antioxidant and mineral supplement was effective in delaying AMD-related vision loss, but was unable to reverse existing vision loss [14,15]. The Age-Related Eye Disease Study (AREDS), sponsored by the National Eye Institute, demonstrated that high levels of antioxidants and zinc were able to reduced the risk of development of advanced AMD by approximately 25% [16]. In 2004, the Lutein Antioxidant Supplementation Trial (LAST) demonstrated that nutritional supplementation with lutein or lutein together with antioxidants, vitamins, and minerals improved visual function and symptoms in patients with atrophic age-related macular degeneration [13].

Nutritional supplements have become the first line of defense for clinicians in battling dry AMD. Vitamin and mineral formulations are a valid therapeutic tool and are many orders of magnitude less toxic than aspirin and acetaminophen [17].

The Taurine, Omega-3 Fatty Acids, Zinc, Antioxidant, Lutein (TOZAL) study sought to identify the potential benefits of a novel supplement designed to limit the risk of AMD and progressive vision loss while also reducing or eliminating the risk of adverse events.

## Methods

This study was approved by an independent ethics committee and an independent review board and was conducted at five independent clinical sites. The study was conducted from 2004 through 2005.

### Study Design

This prospective, double-blind, 6-month trial enrolled 73 subjects with at least 1 eye diagnosed with dry AMD. There were 5 independent study sites: site 1 enrolled 19 patients, site 2 enrolled 15 patients, site 3 enrolled 13 patients, site 4 enrolled 12 patients, and site 5 enrolled 14 patients. Patients were randomly assigned 1 of 2 treatment arms: 1) microcurrent stimulation (MCS) treatment and nutritional supplement (n = 36), and 2) sham MCS and nutritional supplement (n = 37).

In 1998, Allen et al reported that patients with dry AMD treated with a combination of nutrients and microcurrent electricity showed slowing or reversing of the progress of AMD for most subjects [18].

The microcurrent in this study was self-administered by the patient, 2 treatments each day, using an automated microcurrent stimulator with a preset current of 800 micro-amps at frequency settings of 292 Hz (6 minutes), 30 Hz (3 minutes), 9.1 Hz (2 minutes), and 0.3 Hz (1 minute) for a total of 12 minutes. The sham device was identical to the treatment device, including LED indicators and audible tones; however, there was no electrical current output. Electrical current administered at levels below 1 milliamp (1,000 micro-amps) has no detectable sensation.

MCS treatment was found to have little significant effect on any of the efficacy endpoints and thus was abandoned. Only the nutritional supplement aspect of the study is reported and discussed here (ie, patients receiving sham MCS and nutritional supplement, n = 37).

**Figure 1:**
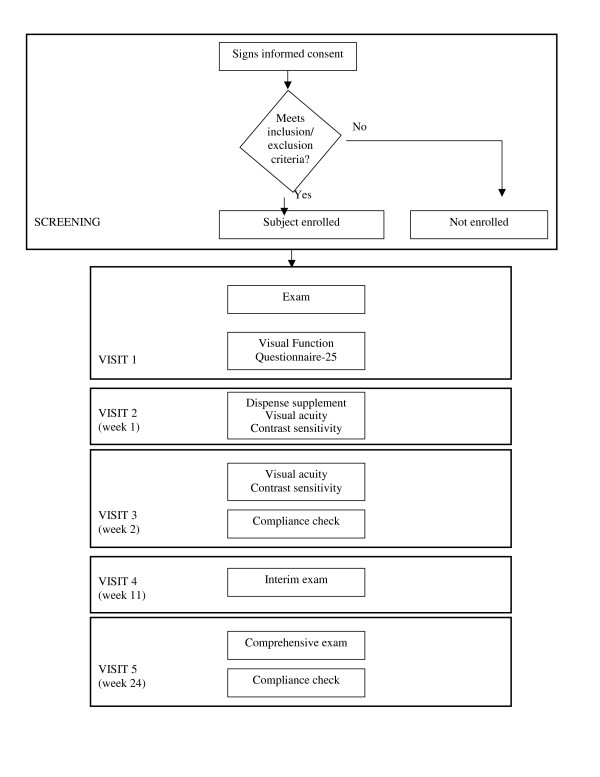
Study design.

Each subject was scheduled for 5 visits (Figure 1). During the first visit, subjects who met the inclusion and exclusion criteria (Table 1) and signed a consent to participate underwent a comprehensive eye examination including medical and ophthalmic history, refraction, BCVA measured by ETDRS (logMAR) at 4 m, biomicroscopy, intraocular lens evaluation, intraocular pressure, dilated fundus exam, fluorescein angiogram and retinal photographs, contrast sensitivity, full threshold visual fields, and macular testing (central 10° threshold visual filed). Additionally, each subject completed the Visual Function Questionanaire-25 (VFQ-25).

**Table 1:** Inclusion and exclusion criteria

Inclusion Criteria
• Signed written consent • Between the ages of 50 and 90, inclusive • Any race or gender • Diagnosis of nonexudative (dry) AMD in at least 1 eye having > 10 large soft drusen 63 μm in diameter, within 3,000 um of the fovea center, documented on macular exam, retinal angiography and fundus photographs • Able to understand and comply with the requirements of the trial • BCVA in the trial eye(s) of 20/32 to 20/125 inclusive as measured by ETDRS (logMAR) • Subjects must not have conditions that limit the view to the fundus (eg vitreous hemorrhage, cataracts, an epiretinal membrane). All subjects with = 2+ nuclear opacities and/or significant central opacity (PSC or ASC) > 1+ will undergo Potential Acuity Meter (PAM) testing. If the vision is = 2 lines improved on PAM over standard acuity measurement then the subject will not be eligible for the trial • Subjects must be available for a minimum trial duration of approximately 6 months • Subjects must agree to take only the nutritional supplement that is provided during this study • Subjects or eyes must not meet any of the exclusion criteria
Exclusion Criteria
Any of the following excluded a subject from the trial: • Currently enrolled in an ophthalmic clinical trial • Eyes with concomitant macular or choroidal disorders other than AMD and with indefinite signs of AMD • Eyes with a diagnosis of exudative (wet) AMD with active subretinal neovascularization (SRNV) or CNV lesions requiring laser photocoagulation in the study eye • Subjects with significant ocular lens opacities causing vision decrease • Subjects with amblyopia • Subjects with optic nerve disease (neuropathy, atrophy, papilledema), unstable glaucoma as defined by intraocular pressures greater than 25 mm Hg, 3 or more glaucoma medications, C/D of 0.8 or greater and visual fields consistent with glaucoma; history of retina-vitreous surgery, degenerative myopia, active posterior intraocular inflammatory disease, chronic use of topical ocular steroid medications, vasoproliferative retinopathies (other than AMD), rhegmatogenous retinal detachment, and inherited macular dystrophies • Subjects with demand type pacemakers or epilepsy • Subjects with uncontrolled hypertension (defined as diastolic of 90 or greater and systolic of 150 or greater) • Subjects with recent history (within the previous year) of cerebral vascular disease • manifested with transient ischemic attacks (TIA's) or cerebral vascular accidents (CVA's) • Subjects with a history of AIDS • Subjects who have received any previous experimental procedure in either eye or the use of any investigational drug or treatment within 30 days prior to enrolling in the trial • Subjects who have had intraocular surgery in trial eye within 3 months prior to enrolling in the trial • Smokers or any tobacco use

During the second visit at week 1, BCVA (logMAR) and contrast sensitivity were measured and the nutritional supplement was dispensed. The TOZAL nutritional supplement formulation used in this study is outlined in Table 2. This is a novel supplement formulation and is currently patent pending. Subjects were instructed to self-administer the oral supplements at 2 capsules 3 times per day concurrent with food intake. Treatment compliance was assessed at each subsequent visit via a daily patient log.

**Table 2:** Nutritional supplement formulation

Component	Weight	Percent of daily value
Vitamin A (total)	28,640 IU	573%
Vitamin A	10,000 IU	
Natural Beta-Carotene	18,640 IU	
Vitamin C	452 mg	753%
Vitamin E	200 IU	667%
Zinc Oxide	69.6 mg	464%
Copper	1.6 mg	80%
Taurine	400 mg	
EPA Omega-3 Fatty Acids	180 mg	
DHA Omega-3 Fatty Acids	120 mg	
Lutein (free, not esterified)	8 mg	
Zeaxanthin	400 mcg	

During the third visit at week 2, BCVA (logMAR) and contrast sensitivity were measured.

During the fourth visit at week 11, BCVA (logMAR) and contrast sensitivity were measured. In addition, retinal photographs, fluorescein angiogram, macular testing, and full threshold visual fields were conducted.

The final and exit visit was at week 24 and was a repeat of the first visit in addition to a compliance assessment.

### Objective Measures

The primary objective was to measure the change in BCVA from baseline to 6 months in subjects with non-exudative macular degeneration treated with a nutritional supplement. The secondary efficacy variable was objective signs of improved macular function.

The primary safety variables were unexpected ocular or systemic findings, adverse event rate, and temporary and permanent discontinuation. Investigators were required to report any treatment-related adverse events or serious non-treatment-related adverse events and severe adverse events requiring hospitalization.

Adverse events included any undesirable clinical occurrence in a subject whether considered related to treatment or not. Serious adverse events included those in which information suggested that treatment caused or may have caused or contributed to death or serious injury including, but not limited to, permanent decrease in BCVA (≥ 2 lines) or hospitalization. Significant adverse events included those that required medical intervention or warranted discontinuation (temporary or permanent) from the clinical trial. These events were non-sight-threatening conditions that were determined to be device-related. Non-significant adverse events were events that did not warrant discontinuation from the clinical trial.

Subjects could discontinue or withdraw from the trial for any reason. Investigators could discontinue a subject if, in his/her opinion, it was in the best interest of the patient, if there was non-compliance with study visits, if there was more that 25% non-compliance with self-administration of treatment, or if there was protocol deviation.

### Placebo Arm

The IRB for this study determined that standard of care for age-related macular degeneration must include an Age-Related Eye Disease Study (AREDS)-type nutritional supplement and that no true placebo arm would be permissible. A placebo arm was constructed from a review of the literature. The exclusion and inclusion criteria used for the Multicenter Investigation of Rheopheresis for AMD (MIRA-1) trial were followed for the TOZAL study. Patient demographics between subjects enrolled in the MIRA-1 study and the TOZAL study were similar. All subjects in the MIRA-1 study received an oral supplement consisting of 400 mg vitamin C, 200 IU vitamin E, 40 mg zinc, and 3,000 IU beta-carotene [7]. The results from the placebo arm of the MIRA-1 study are used as a comparator in this report.

### Statistical Analysis

Sample size and power calculations were based on the primary efficacy endpoint. Results from two rheopheresis studies (Brunner and MIRA-1) were used to estimate the mean change expected [7,19]. Thirty-four patients were included in the per-protocol analysis.

Statistical analyses were performed using SPSS for Windows (SPSS 14.0, SPSS Inc., Chicago, IL). A paired t-test was used to test the null hypothesis, with the average VA score the same at baseline and follow-up. A two-sided alpha level of 0.05 was used to determine statistical significance.

Primary efficacy endpoint analysis consisted of all randomized and dispensed subjects with baseline and at least 1 post-treatment VA recorded. Baseline was equated to VA measured as visit 2 (week 1). Change from baseline was evaluated at weeks 11 and 24. Repeated measures mixed model (Proc Mixed, SAS, 8.2) was fitted to compare mean change in VA from baseline between the groups, with Visit and Treatment-by-Visit interaction included as main effects, baseline VA as covariate, and Eye as the within-patient random effect.

A secondary analysis set was also constructed, comprised of 1 eye per patient meeting the 20/32–20/125 entrance VA criteria. If both the left and the right eye met the criteria, the "best" eye was used.

The safety analysis set consisted of all randomized subjects who received at least 1 dose of treatment. Incidence of unexpected ocular or systemic findings, adverse events, and temporary/permanent discontinuation were tabulated and evaluated using Fisher's exact test. All tests were carried out at α = 0.05, 2-sided.

## Results

### Demographics and Baseline Characteristics

Demographics and baseline characteristics of the 37 patients receiving the nutritional supplement are outlined in Table 3. Subjects enrolled in the TOZAL study were matched for inclusion and exclusion criteria with the MIRA-1 study. Subjects in both cohorts were similar across age, gender, ethnicity, and mean baseline BCVA [7].

**Table 3:** Demographics and baseline characteristics

Variable	Parameter	Treatment Group (n = 37)	Placebo Group (n = 15)
Gender, n (%)	Female	20 (54.1)	10 (67.0)
	Male	17 (45.9)	5 (33.0)
Age	Mean ± SD	76.3 ± 7.8	74.7 ± 5.9
	Range	54 to 90	66 to 85
Ethnicity, n (%)	African American	1 (2.7)	0 (0)
	Asian	1 (2.7)	0 (0)
	Caucasian	34 (91.9)	15 (100)
	Hispanic	1 (2.7)	0 (0)
Current smoker, n (%)	No	37 (100)	-
Former smoker, n (%)	No	25 (67.6)	-
	Yes	11 (29.7)	-
	Yes, 27 years ago	1 (2.7)	-
Family history of MD, n (%)	Yes	9 (24.3)	-
Diabetes	Yes	4 (10.8)	-
Hypertension	Yes	16 (43.2)	-
Heart Disease	Yes	13 (35.1)	-
Other	Yes	31 (83.8)	-
Cataract surgery	Yes	31 (83.8)	-
Refractive surgery	Yes	0 (0)	-
Glaucoma	Yes	8 (10.8)	-
Diabetic retinopathy	Yes	0 (0)	-
Mean baseline BCVA (logMAR)	Mean ± SD	0.41 ± 0.17	0.39 ± 0.17

### Visual Acuity Outcomes

**Figure 2:**
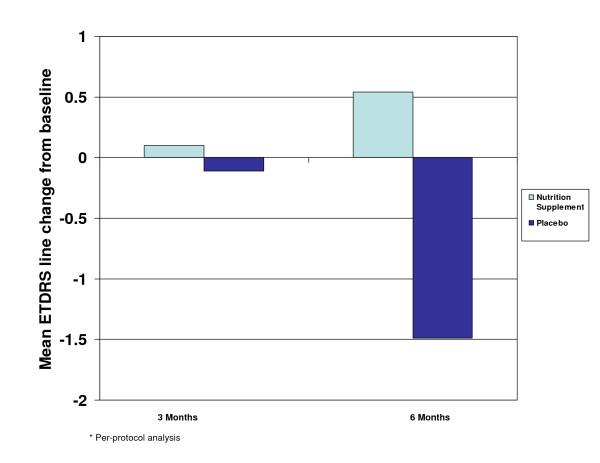
Mean ETDRS line change at 3 and 6 months.

In the per-protocol analysis, the mean change from baseline in ETDRS BCVA (logMAR) was calculated at 3 and 6 months (Figure 2). While the placebo arm experienced a negative mean ETDRS line change of 1.49 lines at 6 months (loss of VA), the treatment group demonstrated a positive mean ETDRS line change of 0.54 lines at 6 months (gain in VA). The mean logMAR line difference between the treatment and placebo-control groups was 2.03 lines at 6 months postbaseline. A continual improvement in BCVA (logMAR) over time was demonstrated in the treatment group, while overall, the placebo arm continued to lose VA over time.

**Figure 3:**
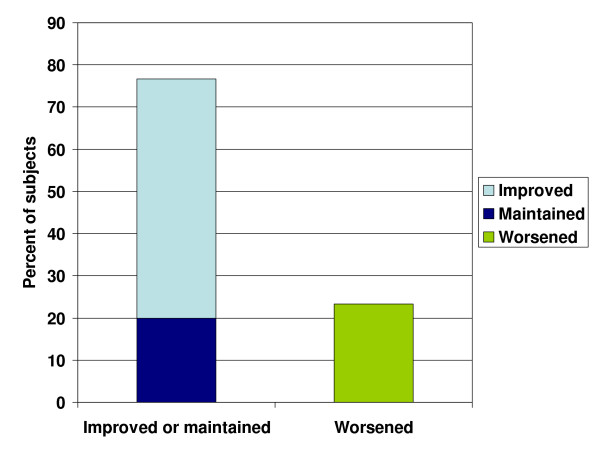
Percent of subjects with improved or maintained BCVA at 6 months.

At 6 months, of those subjects in the treatment arm, 56.7% experienced improved BCVA (logMAR), 20.0% maintained their BCVA (logMAR), and 23.3% experienced worsened BCVA (logMAR). Overall 76.7% of patients improved or maintained their BCVA (logMAR) with the TOZAL nutritional supplementation (Figure 3).

### Secondary Outcomes

Fluorescein angiogram, retinal photographs, contrast sensitivity, full-threshold visual fields, macular testing (central 10° threshold visual field), and the Visual Function Questionanaire-25 (VFQ-25) were found to have little significant change at 6 months.

### Statistical Analyses

**Figure 4:**
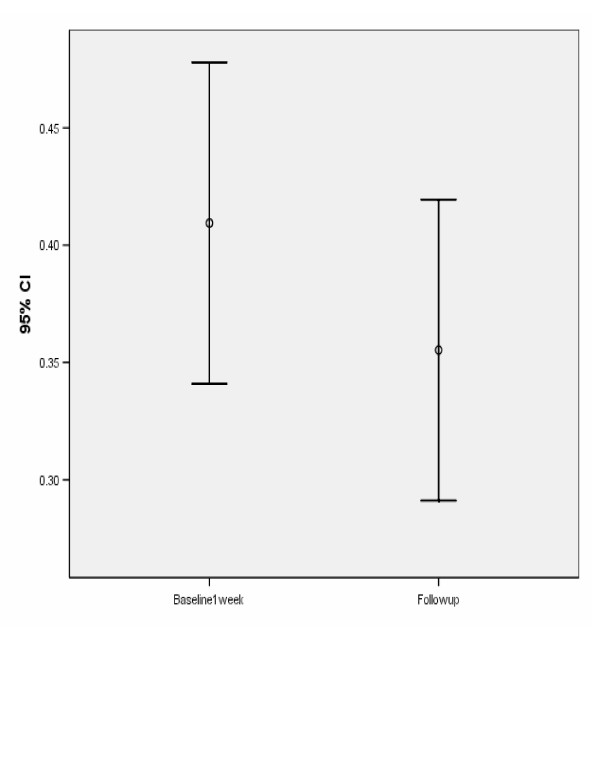
Average (and 95% CI for average) visual acuity at baseline and at follow-up.

The average (SD) VA score was 0.409 (0.196) versus 0.355 (0.184) for baseline and follow-up respectively, t = -2.09; df = 33; *P *= .045. Figure 4 demonstrates the average (and 95% confidence interval [CI] for the average) VA score at baseline and at follow-up. Tables 4 and 5 show a statistically significant increase in the VA score from baseline to follow-up. Thus, the null hypothesis, the average VA score is the same at baseline and follow-up, was rejected and it was concluded that there was a statistically significant improvement in VA from baseline to follow-up. Table 5 shows that the average increase in VA was 0.0541 and the 95% CI for the average increase was (-0.107, -0.0013).

**Table 4:** Paired samples statistics

		Mean	N	Std. Deviation	Std. Error Mean
Pair 1	Baseline	.409	34	.1963	.0337
	Follow Up – 6 months	.3553	34	.18384	.03153

**Table 5:** Paired samples test

		Paired Differences							
					
					95% Confidence Interval of the Difference				
								
		Mean	Std. Deviation	Std. Error Mean	Lower	Upper	t	df	Sig. (2-tailed)
Pair 1	Follow Up -Baseline	-.05412	.15132	.02595	-.10692	-.00132	-2.085	33	.045

A post-hoc power analysis reveals that a sample size of 34 achieves 80% power to detect a difference of 0.062 between the baseline and follow-up average acuity score assuming a standard deviation of the differences (follow-up minus baseline) of 0.126 and with a significance level (alpha) of 0.05 using a two-sided paired t-test. If the true population change (follow-up minus baseline) was -0.062 with a standard deviation of 0.126, then this study would have had an 80% chance of detecting this difference at the 0.05 level of significance.

### Adverse Effects

There were no significant systemic or ocular adverse events related to the nutritional supplement. The most frequent events were systemic gastrointestinal (GI) reactions, including gastric upset, reflux, nausea, and taste perversion. The majority of these events occurred in patients who had not followed the prescribed procedure of taking the supplement concurrent food intake. After adjusting their treatment schedule to always administering the nutritional supplement with food, the majority of GI issues resolved. Overall, there does not appear to be any significant adverse events related to the nutritional supplement.

## Discussion

### Visual Acuity

Left untreated, patients with AMD are at risk for substantial vision loss. The literature suggests that without intervention, patients with AMD will experience a loss in VA of at least 0.8 lines and up to 1.5 lines at 6 months [5-7].

The LAST study reported Snellen equivalent VA improvements in both intervention groups (Group 1 L: lutein 10 mg; Group 2 L/A: lutein 10 mg/antioxidant/vitamin and mineral broad-spectrum supplementation formula), with mean eye improvements of 5.4 letters for group 1 L (95% CI, 2.7–8.0, *P *= .01) and 3.5 letters for group 2 L/A (95% CI, 0.8–6.1, *P *= .04) [13].

The 6-month LUXEA study demonstrated that supplementation with carotenoids, lutein, and zeaxanthin can improve mesoptic contrast acuity thresholds and visual performance at low illumination [20].

The CARMIS study treated 153 patients with AMD (AREDS category 3, 4) and VA of greater than 20/32 (0.3 LogMAR) with lutein 10 mg, zeaxanthin 1 mg, astaxanthin 4 mg, vitamin C 180 mg, vitamin E 30 mg, zinc 22.5 mg, and copper 1 mg (AZYR SIF^®^, Sifi Italy). Patients received baseline, 6-month, and 1-year follow-up with ETDRS and the 39-item NEI-VFQ. After 1 year, treated patients showed stabilization of VA and significantly better ETDRS scores (87 +/- 6) compared to controls (80 +/- 7; *P *= .02). VFQ-39 scores were significantly increased in the treatment group (*P *= .001) [21].

The AREDS formula does not address the vast majority of patients who want to improve visual function while preventing advanced AMD. The targeted nutritional supplement prescribed in the TOZAL trial allowed for 76.7% of subjects to improve or maintain their VA, with up to 0.5 lines of VA improvement at 6 months.

### Nutritional Supplements

Countless studies support the use of high-dose vitamins, antioxidants, omega-3 fatty acids, zinc, and carotenoids in the treatment of AMD. In addition, studies on the serum levels of compounds including vitamins A, C, and E, carotenoids, zinc, selenium, and fibroblast growth factor in subjects with AMD suggest that low levels of these compounds put patients at greater risk for the development of AMD [8-13]. However, recent reports of adverse events associated with specific supplement components emphasize the need for improved supplement formulation.

Results of several large studies suggest that supplemental beta-carotene increases the risk of developing lung cancer in heavy smokers [22-24]. Thus, it has been recommended that subjects with a history of smoking avoid supplemental beta-carotene as part of an AMD prevention program. However, an increased intake of foods rich in beta-carotene has not been found to pose a heightened risk for the development of lung cancer among current and non-smokers [25]. Other carotenoids derived from whole foods (lutein, zeaxanthin, and lycopene) are also not associated with increased risk for lung cancer [25].

The supplement prescribed in the TOZAL study was designed to address the risk of lung cancer among smokers receiving supplemental beta-carotene by focusing on beta-carotene derived from whole foods. The TOZAL supplement contained 18,640 IU of natural beta-carotene and 10,000 IU of vitamin A.

Recent data link high doses of vitamin E to a 13% increase in the risk for heart failure [26]. In addition, a separate study found that doses of 400 IU or more of vitamin E increased the chance of early death or, according to the [23,24] authors, "all-cause mortality" and should be avoided [27]. In an attempt to address these potential risk factors, the TOZAL supplement was designed with 200 IU vitamin E.

Supplemental zinc has been found to decrease the rate of loss of VA associated with AMD [16]. High doses of zinc were included in the AREDS supplement (80 mg as zinc oxide), as well as copper (2 mg) to help prevent copper deficiency associated with zinc supplementation. In the AREDS study, 7.5% of participants receiving a zinc-containing nutritional supplements vs 5.0% of participants receiving no zinc in their nutritional supplement reported urinary tract problems that required hospitalization, as well as increased rates of anemia (anemia results were found not to be statistically significant) [16]. In an effort to limit the adverse effects associated with high-dose zinc, the TOZAL supplement was designed with 69.6 mg zinc and 1.6 mg copper. No urinary tract adverse events or anemia were reported during the TOZAL trial.

In 1998, van den Berg et al found that lutein negatively affected beta-carotene absorption when the two were given simultaneously [28]. This decrease in absorption may affect the amount of beta-carotene available for conversion to retinol. In the AREDS study, lutein was not a constituent of the formula, thereby circumventing the issue. As newer formulas are developed that contain lutein, consideration must be given to administering vitamin A in addition to beta-carotene. In the TOZAL study, this adjustment was made by administering 18,640 IU of natural beta-carotene and 10,000 IU of vitamin A.

While toxicity has occurred at dosages of vitamin A of up to 50,000 IU/day for a period of 18 to 24 months, [29-32] an intake of 10,000 IU/day has not been associated with toxicity and is considered safe.

## Conclusion

The present study confirms previously published reports on the direction and magnitude of improved visual acuity in dry AMD [13,20,21]. That we did not find improvements in other visual function parameters such as the contrast sensitivity function, is likely related to the short duration of this study. The National Eye Institute's AREDS II trial will follow a qualitatively similar supplement (i.e., the AREDS II formula has lower zinc, higher omega-3, lutein and zeaxanthin in some of its treatment arms) as TOZAL (without the addition of taurine). The results of the TOZAL study reported here support the potential for positive visual outcomes in the AREDS II trial.

We can not completely dismiss microcurrent stimulation (with or without supplementation) as we did not evaluate all available methods of stimulation.

Treatment based on dietary manipulation should continue to be pursued and refined as a simple, low-cost, effective therapy for AMD.

## Competing interests

The author(s) declare that they have no competing interests.

## Additional files 1,2

## Pre-publication history

The pre-publication history for this paper can be accessed here:



## Supplementary Material

Additional File 1 TOZALv2_database Click here for file

Additional File 2 TOZALv2_database Click here for file
